# The American Agriculturist

**Published:** 1878-12

**Authors:** 


*The American Agriculturist.*

Many times the small cost will be returned to every person, in the country, or village, or city, who supplies himself and family with the plain, practical, reliable, useful, *paying* information given in the *American Agriculturist.* It was so named because started 37 years ago as a rural journal, but is now greatly enlarged in size and scope, and profusely illustrated, so that it meets the wants of all classes—of cultivators of the smallest plots, or of the largest farms—of Housekeepers and Children—of owners of Cattle, Horses, Sheep, and Swine of Fruit Growers, Florists, Builders, Mechanics, etc. From 600 to 800 original Engravings in every volume, bring right to the eye and understanding, many useful, labor-helping and labor-saving contrivances, largely home-made, and for out-door and in-door work; also plants, animals, construction of dwellings, etc., etc These numerous Engravings make this Journal greatly superior to every other one treating on the same subjects. The persistent, caustic exposures of Humbugs and Swindles are of great value to all its readers.—Over $25,000 a year are expended in collecting useful and interesting information and engravings, the benefit of all which can be enjoyed at the reduced price of only $1.50 a year, post-free; or four copies at $1.25 each, or ten copies at $1 each. A specimen copy, 10 cents. Try it a year. It will pay. Published by Orange Judd Co., 245 Broadway, New York.

N. B.—A copy of Marshall's magnificent Steel Plate Engraving, "The Farmer's Pride," is delivered free to every subscriber of the *American Agriculturist* who sends 20 cents extra to cover cost of packing and postage.

Also, *The Examiner* will be greatly improved during the ensuing year. It will furnish all the local news, notices of meetings, assessments, tax and other sales, markets, etc , etc., giving information that every resident of this County *ought* to have, and cannot deprive himself of, without being likely to lose many times the cost of the paper. The Village, Farm, Town and County Talk on subjects of local interest, will be supplied fully in our colums, besides much interesting reading. All this can be had at scarcely 4 cents a week, or only $2 for the entire year!

